# Simultaneous Lateral Interbody Fusion and Posterior Percutaneous Instrumentation: Early Experience and Technical Considerations

**DOI:** 10.1155/2015/458284

**Published:** 2015-11-16

**Authors:** Doniel Drazin, Terrence T. Kim, J. Patrick Johnson

**Affiliations:** ^1^Department of Neurosurgery, Cedars-Sinai Medical Center, Los Angeles, CA 90048, USA; ^2^Department of Orthopedic Surgery, Cedars-Sinai Medical Center, Los Angeles, CA 90048, USA; ^3^Department of Neurosurgery, UC Davis Medical Center, Sacramento, CA 95817, USA

## Abstract

Lumbar fusion surgery involving lateral lumbar interbody graft insertion with posterior instrumentation is traditionally performed in two stages requiring repositioning. We describe a novel technique to complete the circumferential procedure simultaneously without patient repositioning. Twenty patients diagnosed with worsening back pain with/without radiculopathy who failed exhaustive conservative management were retrospectively reviewed. Ten patients with both procedures simultaneously from a single lateral approach and 10 control patients with lateral lumbar interbody fusion followed by repositioning and posterior percutaneous instrumentation were analyzed. Pars fractures, mobile grade 2 spondylolisthesis, and severe one-level degenerative disk disease were matched between the two groups. In the simultaneous group, avoiding repositioning leads to lower mean operative times: 130 minutes (versus control 190 minutes; *p* = 0.009) and lower intraoperative blood loss: 108 mL (versus 93 mL; NS). Nonrepositioned patients were hospitalized for an average of 4.1 days (versus 3.8 days; NS). There was one complication in the control group requiring screw revision. Lateral interbody fusion and percutaneous posterior instrumentation are both readily accomplished in a single lateral decubitus position. In select patients with adequately sized pedicles, performing simultaneous procedures decreases operative time over sequential repositioning. Patient outcomes were excellent in the simultaneous group and comparable to procedures done sequentially.

## 1. Introduction

Spinal fusions were conventionally done through open surgical methods via anterior or posterior approaches. With the recent advancements in technology, surgical methods, and imaging techniques, innovative minimally invasive spine surgery has emerged [1–3]. A recent popular development has been the lateral transpsoas approach, which became a reality through enhanced visual ability, improved retraction techniques, and a better understanding of surgical anatomy [1–3]. While this modern technique offers multiple advantages, surgeons should understand its methodology, indications, and possible complications. Introduced by Mayer in 1997 and later modified by McAfee, Pimenta, and Ozgur, the procedure approaches the lumbar spine laterally through retroperitoneal fat and the psoas major [4–6]. Problems are occasional because it evades the major blood vessels and abdominal organs, and it eliminates the need for another surgeon for anterior access [6].

Lateral interbody lumbar fusion procedures are becoming more common for degenerative lumbar disease requiring instrumentation and they are typically performed by repositioning the patient to complete the second stage of the circumferential procedure [7, 8]. We have developed a method for performing both procedures in a single lateral position, which may shorten the length of surgery and increase operative efficiency while maintaining surgical precision.

The following technique should be considered in select patients undergoing a minimally invasive lateral transpsoas interbody fusion with a subsequent posterior percutaneous instrumentation. This technical note describes our early experience with this method, our technical nuances, and the potential benefits that it may provide for both the surgeon and the patient.

## 2. Preoperative Considerations

### 2.1. Patient's Bony Anatomy and Body Habitus

With normal anatomic variances among spine patients, we would tend to avoid this technique in “small or atrophic/dystrophic” pedicles, “rotated, asymmetric” pedicles, or certain L5 pedicles that are difficult to visualize. Additionally, on severe osteoporotic patients with difficult visualization of pedicle anatomy, it is not recommended. Although MIS techniques can be used effectively in larger obese patients, our technique is limited by the fluoroscopic penetrance through soft tissue—which can affect clear visualization of the bony anatomy. On the other hand, in patients with multiple severe comorbidities patients who require several personnel to assist for repositioning, we find that this is a strong consideration for application of this technique.

### 2.2. Number of Levels of Instrumentation

In patients with more than two levels of instrumentation, we have found unique limitations in the instrumentation systems, difficulty with pedicle cannulation, and complexity of rod delivery. As we find no appreciable gain/efficiency in operative time saved, we do not recommend this technique in patients beyond two levels of instrumentation.

Complication avoidance with simultaneous lateral interbody fusion and percutaneous posterior instrumentation in the lateral position consisted in the following:

 Avoiding small, atrophic/dystrophic, rotated, asymmetric pedicles.
 Avoiding morbidly obese if using fluoroscopy.
 Limiting the procedure to two levels of instrumentation.

## 3. Operative Considerations

### 3.1. Selection of Operating Room Table

Table selection and patient positioning are crucial to the success of this surgical technique. Selection of the ideal operating table is typically one with multiplane adjustment capabilities for use after the patient is secured to the table (Sliding Skytron or breakable Jackson table). In our practice, we utilize a sliding Skytron table with a kidney bump. Before the patient is transferred to the operative table, a “reverse” of the orientation is made so the foot of the table is at the head anesthesiologist location. The table is also slid to the farthest extent to keep the base of the table at the anesthesiologist head—away from the operative field. This allows easy introduction of the mobile fluoroscopy unit and seamless transition between the AP and lateral planes.

### 3.2. Patient Positioning

Once the patient is turned in the lateral decubitus position, an axillary roll is inserted and all pressure areas are padded. The arms are positioned away from the abdomen, and the patient's thorax and pelvis/lower extremities are tapped to the table—aligning the greater trochanter to the break in the table. The table is then adjusted using fluoroscopic guidance to achieve the ideal visualization for the disc spaces and bony pedicles.

A specific note is made to placement of the lateral decubitus patient beyond the lateral edge of the table as much as possible (towards the surgeon's side). The dorsal lumbar soft tissue should be hanging over the lateral table and beyond a line drawn from the lateral edge of the OR table. This technical pearl is emphasized in order to achieve enough room for a lateral-medial trajectory for cannulation of the pedicles closest to the floor. We have found that if the patient's dorsal spine does not adequately hang over the lateral edge of the operative bed, then potentially the surgeon will be blocked by the OR table/mattress leading to a potentially unwanted lateral-based screw trajectory.

The lateral flank and posterior spine are prepped and draped in one contiguous fashion. Oftentimes, two iodine-impregnated adhesive skin barriers are required. A note is made to completely prep and drape out the entire dorsal posterior lumbar spine.

### 3.3. Specific Techniques: Transpsoas Lateral Interbody Fusion

To localize the incision, a perfect lateral fluoroscopic image is obtained centered over our desired disc space. Previous reports have identified ideal starting points for each lumbar disc in relation to the lumbar plexus anatomy [8]. A single oblique incision is made, and a combination of blunt and electrocautery dissection is made to the level of the oblique muscle fascia. The oblique and transversalis muscles are bluntly dissected without electrocautery to the deep abdominal/transversalis fascia. A sharp incision is made through the fascia under direct visualization with care not to pass point, and the retroperitoneal space is entered. Confirmation of the correct anatomic space is made with digital palpation and direct visual inspection. The retroperitoneal space is developed by sweeping the peritoneal contents ventrally, palpating the bony transverse process dorsally, palpating the ilium/iliac wing caudally, and identifying the belly of the psoas muscle deep. The opportunity to “shallow dock” or utilize a microscope for visualization is possible; however, this is not commonly employed in our practice.

A neuromonitoring electrical stimulation probe is guided down to the psoas with finger retraction of the peritoneal contents. A lateral fluoroscopic image is used to guide our probe trajectory. With free-running EMGs, the stimulated probe is used to traverse the psoas muscle and enter the disc space. Care is taken not to injure the lumbar plexus or associated nerve roots. Serial dilators and specialized illuminated retractors are deployed and the discectomy and interbody insertion are completed. Throughout the procedure neurophysiologic electromonitoring is utilized.

### 3.4. Specific Techniques: Percutaneous Posterior Instrumentation and Fusion

**Figure 1:**
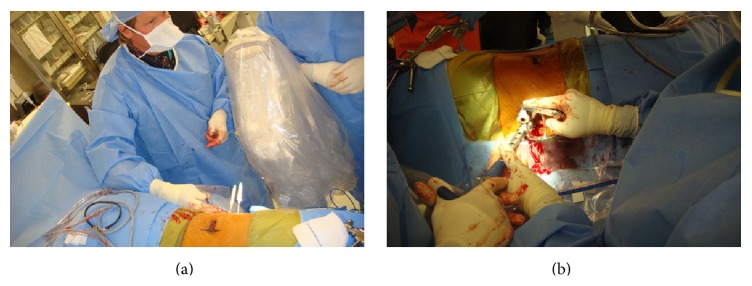
Intraoperative photographs of Jamshidi needle placement (a) followed by posterior fixation (b) while in the lateral position.

The patient position remains* unchanged* for the second-stage posterior procedure. Implant selection for this technique is helpful as instrumenting a lateral decubitus patient requires an additional level of difficulty and spatial orientation. Recommendations are made to utilize MIS instrumentation systems that allow for “guided” insertion of the rod to the pedicle screw reduction towers. In our experience, for one- to three-level fusions, guided MIS rods (MIS Sextant, Medtronic Inc., Memphis, TN, and MIS Ballista, Biomet Inc., Warsaw, IN) offer the ability to insert percutaneous rods into pedicle screw reduction towers with ease and without significant soft tissue difficulty (Figures 1(a) and 1(b)). We recommend, however, any system with which the surgeon feels most comfortable.

Percutaneous delivery of posterior instrumentation is done in the standard fashion utilizing fluoroscopic guidance, Jamshidi trocar, Kirchner guide wires, and cannulated instruments. Traditional MIS techniques involving a lateral-based insertion point away from the adjacent facet joint, correlation with acceptable AP/lateral imaging, and confirmatory neuromonitoring stimulation of pedicle taps are employed. It is noted once again that because the patient is positioned beyond the lateral edge of the operative table, insertion of the ideal “lateral to medial” trajectory of the pedicles closest to the floor is uninhibited. We do confirm that there is an initial learning curve with instrumenting a laterally positioned patient and report the threshold for comfortability to be at approximately 8–10 cases.

The rods are inserted in a standard fashion either through a separate or previous incision and all set screws are final tightened to manufactured settings. All instrumentation is removed and the wounds are irrigated and closed in a standard fashion.

### 3.5. Intraoperative CT-Guided Navigation of Instrumentation

We have most recently replaced the use of fluoroscopy for posterior percutaneous instrumentation with intraoperative CT-guided spinal navigation for patients requiring more than one-level fusions. In this lateral position, we have found no significant limitations in interchanging imaging modalities. Additionally, we have found similar results in regard to workflow efficiency, OR time, and clinical outcomes. However, this remains outside of the full scope of this paper.

## 4. Methods

We performed a retrospective chart review of ten consecutive patients who underwent both procedures simultaneously (nonrepositioned) and compared the outcomes with a control group of ten patients who underwent the lateral interbody fusion and were then repositioned for posterior percutaneous screw fixation (repositioned). Across both groups, patients were matched for by age and reason for surgery. In both groups, pars fractures with instability (4), mobile grade two spondylolisthesis (4), and the remaining patients had severe degenerative disk disease at a single level (12). Indications for surgery included worsening back pain in patients who failed exhaustive conservative management.

## 5. Illustrative Case

**Figure 2:**
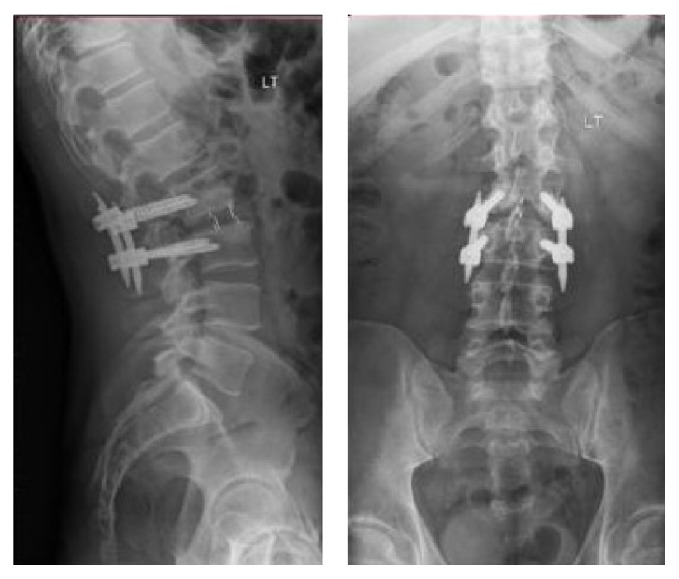
Postoperative lateral and AP views showing lateral interbody graft and posterior instrumentation.

A 47-year-old male presented with end-stage degenerative disc disease at L2/3 with associated severe back pain and bilateral lower extremity radiculopathy that had failed an extensive course of nonoperative management. Advanced imaging, including CT and MRI, revealed a grade 1 degenerative spondylolisthesis with associated facet arthrosis. Due to the collapse of the disc space, MRI demonstrated lateral recess and foraminal narrowing with neural stenosis but no acute herniated nucleus pulposus or severe ligamentum hypertrophy. Intraoperatively, the patient was placed in a right lateral decubitus position with the left flank and posterior dorsal spine prepped and draped in one setting. He underwent an L2/3 left lateral transpsoas discectomy and interbody fusion, followed by posterior percutaneous fixation without repositioning. Postoperative films were adequate (Figure 2), and he experienced no intraoperative or clinical complications.

## 6. Results

**Figure 3:**
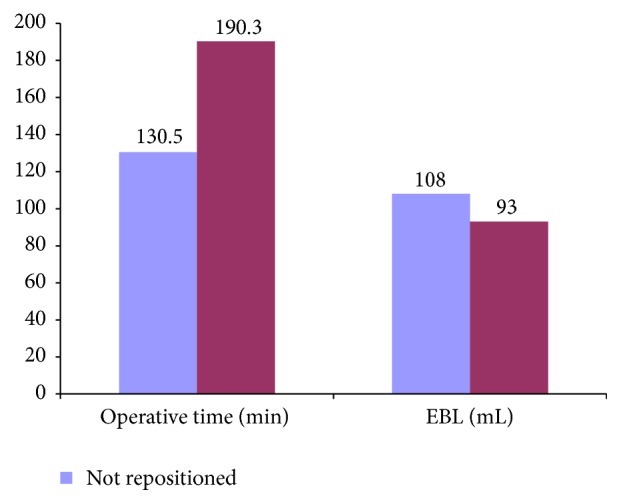
Graph of estimated blood loss and operative time in repositioned versus nonrepositioned patients.

Between March 2010 and November 2011, twenty patients underwent lateral interbody fusion followed by posterior percutaneous screw fixation followed for an average of 9 months (range 6 months–12 months). The nonrepositioned group included 3 women and 7 men, while the repositioned group included 6 women and 4 men. The average age was 54.5 years (range 30–78, nonrepositioned) and 57.8 years (range 45–71, repositioned). Avoiding repositioning, operative time from incision to closure averaged 130.5 minutes (versus repositioned 190.3 minutes; *p* < 0.05) and intraoperative blood loss was 108 mL (versus 93 mL; NS, Figure 3). Nonrepositioned patients were hospitalized for an average of 3.8 days (versus 4.1 days; NS). Details of patients' characteristics are outlined in Table 1. Of the twenty patients who underwent surgery, there were 3 patients (2 in repositioned and 1 in nonrepositioned) who experienced transient (less than 2 weeks) postoperative numbness on the side they had the lateral interbody fusion. No patient, in either group, reported weakness or significant pain related to the approach from the postoperative period through the most recent follow-up. Postoperative imaging confirmed appropriate positioning of the hardware; however one patient in the repositioned cohort required screw repositioning.

## 7. Discussion

During the earliest years, surgeons operated on the spine through the most direct approach to reach the vertebral column—via a posterior fashion [1]. With the emergence of Pott's disease, anterior approaches were introduced to combat this vertebral infectious process [1]. The focal point of both approaches was reestablishing spinal biomechanical stability. Compared to posterolateral fusions, interbody fusions have been theorized to provide finer alignment, greater rates of fusion, and superior patient results [9–13]. An anterior lumbar implant has been shown to contribute significantly to biomechanical durability [1], and implant placement techniques have advanced through ALIF, PLIF, TLIF, and laparoscopic ALIF to the more recent lateral interbody fusion (LIF).

Reported by Burns in 1933, the anterior lumbar interbody fusion (ALIF) was one of the first lumbar interbody surgeries performed [1, 3, 14]. As an anterior approach for the treatment of spondylolisthesis, a complete discectomy was performed and a cadaveric bone graft was utilized [1, 3, 14]. The ALIF delivers a straight approach to the disc space with perhaps the greatest exposure providing the capability to achieve a more complete discectomy and fusion [3, 9, 15–17]. Additionally, there is no nerve root retraction or intrusion into the spinal canal as seen with the PLIF [15, 18]. The utility of the procedure has expanded to treat a number of spine conditions, including neoplastic conditions, infectious process, deformities, and instability [3, 18–21]. Disadvantages are serious and include the possibility of damage to anterior vessels, abdominal organs, sympathetic nerve plexus, and retroperitoneal structures [1, 3]. Moreover, if supplemental posterior instrumentation or decompression is required, a separate posterior incision and approach must be performed. The technique was modified posteriorly as a posterior lumbar interbody fusion (PLIF) in 1953 by Cloward with the goal of maintaining facet joints along with a cage or graft [2, 3, 22]. The PLIF permitted a thorough decompression of the nervous structures without disrupting abdominal elements. At the same time, it allowed the surgeon to perform a more circumferential fusion [1, 3]. Ntoukas and Müller compared results of the traditional “open” PLIF with a percutaneous minimally invasive PLIF and found the percutaneous approach to have less mean blood loss (135 versus 432 mL) and a shorter hospital stay (5 versus 10 days) but a longer operative time (275 versus 152 min) without significant difference in clinical and radiological outcome [23]. A disadvantage of the technique is that it entails retraction of nerve roots to permit sufficient discectomy potentiating nerve root and dural injuries, a hurdle that the transforaminal lumbar interbody fusion (TLIF) would attempt to counter later in the 1980s [1, 3, 9].

In the years following the introduction of the PLIF, reports about the ALIF's surgical strain and patient morbidity began to emerge [1, 24]. This would be the impetus later in 1990s for minimally invasive variations on the operation such as laparoscopic and mini-open approaches. But prior to these developments, an essential procedure was introduced in the 1980s: the TLIF. As mentioned, the TLIF tried to counter hurdles of its PLIF predecessor and additionally offered a better view of the disc space [1, 3, 9]. Since it is a posterior approach, spine surgeons were more comfortable with it, allowing them to correct posterior pathological processes and perform more complete fusions [25–27]. It also avoids any contralateral damage and thus decreases the likelihood of future complications in neighboring levels [3, 28–30]. In a prospective study of 52 patients undergoing the procedure, Hackenberg et al. [31] reported an average operative time of 173 minutes, an average blood loss of 485 mL, a fusion rate of 89%, and 4 (7.7%) patients developing serious complications. Unlike other approaches, the extent of discectomy that can be performed is limited with the TLIF [9]. Additionally, similar to the PLIF, the TLIF removes various components of the posterior structures compromising stability, can cause spinal canal scarring, and can damage paraspinal muscles [3, 21]. Nonetheless, the evolution in the way lumbar interbody fusions were performed up to this point would provide the framework for the development of the lateral transpsoas procedure introduced in 1997.

The 1990s saw pioneering alterations to lumbar spine fusion methods. Indeed, these new techniques would catapult spine surgery into the minimally invasive surgical era. In the earlier part of the decade, the laparoscopic and mini-open modifications of the ALIF were introduced [1–3]. While they were deemed as safer alternatives, they still had similar risks as the original, such as great vessel harm and retrograde ejaculation [1, 3, 6]. There were also the new obstacles of becoming familiar with laparoscopic tools, CO_2_ gas insufflation, and possibility of bowl perforation [1, 32]. Additionally, the learning curve for some of these elaborate techniques seemed to be quite high [4, 6]. Over the next few years, significant improvements in retraction tools and advances in instrumentation would further the application of minimally invasive surgical techniques to the lumbar spine. McAfee was first to report on an anterolateral retroperitoneal approach for treatment of thoracolumbar fractures in the mid-1980s [1, 33]. Key players in lumbar spine surgery would later apply minimally invasive techniques to this operation during the mid-1990s.

In 1998, McAfee et al. published on a minimally invasive microsurgical retroperitoneal lateral approach that would lay the foundation for the direct lateral interbody fusion [4]. With a 4 cm skin incision, muscle-splitting exposure, a self-retaining spreader frame, and microscopy, the technique was revolutionary. The procedure minimized surgical trauma to the patient, used standardized surgical instruments, emphasized good illumination along with the surgical microscope, had minimal blood loss (67.8–168 mL), and decreased operative time (2.0–2.25 hours) [4]. Importantly, spine surgeons could easily implement this technique as it did not necessitate the learning of methods that were completely foreign to them, nor did it dictate the type of fusion [4, 34]. For patients, it provided minimal surgical discomfort, decreased postoperative morbidity, and improved recovery time [4, 34]. Problems are occasional and primarily involve temporary paresis and dysesthesias in the lower extremities [2, 35]. Shortly thereafter, McAfee reported a similar procedure that utilized endoscopy, balloon insufflator dissection, and placement of interbody cages [5]. Compared to the traditional anterior approach, the technique also had lower morbidity, mean length of hospital stay (2.9 days), mean blood loss (205 cc), and operative time (115.2 minutes). Additionally, there were no cases of pseudoarthrosis or implant migration at mean follow-up of 24.3 months. Ahmadian et al. would next describe what became known as the extreme lateral interbody fusion, which had tremendous advantages over conventional anterior and posterior methods [6].

The extreme LIF was initially presented by Pimenta in 2001 as a variation on the retroperitoneal approach to the lumbar spine [2, 6]. It provides similar advantages to its predecessors including a gentle learning curve, lack of necessity of an access surgeon, and a reduction in complications such as visceral harm, great vessel injury, and sexual dysfunction [3, 6]. Aside from this, it also preserves the posterior ligaments and bony structures and thus maintains anatomical stability and alignment while providing maximal access to the disc space and ring apophysis to allow for a complete disc extrusion and deformity adjustment [1]. It does not require retraction or distention of the psoas major and thus the likelihood of transient paresthesia and paresis due its injury are diminished compared with prior lateral approaches [6]. As with prior minimally invasive procedures, the extreme LIF is highly fluoroscopy dependent, which in return is operator and surgeon dependent [6]. As with all lateral fusions, there is also a risk of injury to the genitofemoral nerve and lumbosacral plexus, which has been cited as the most common complication [7]. Moreover, for patients that require percutaneous pedicle screws, repositioning is required to the prone position or a staged procedure is done [6]. Our new novel procedure, explained in detail in this paper, does not require repositioning or a staged procedure, and screw placement is done simultaneously in the same lateral transpsoas position.

The minimally invasive retroperitoneal transpsoas lateral approach is becoming more popular among spine surgeons [7, 34]. Since its introduction, the technique has been applied to treat multiple spinal disorders including degenerative lumbar disease, spondylosis, spondylolisthesis, deformity, trauma, infection, and tumor [34]. As such, more and more LIFs are performed along with posterior percutaneous screw fixation. Typically, such a circumferential procedure is done by repositioning the patient to complete the second stage of screw placement. In order to avoid the problem of repositioning, we have developed a method for performing both procedures in a single lateral position. This technique will shorten the length of surgery and increase operative efficiency while maintaining surgical precision. We have shown that the procedure is applicable to a number of spinal conditions including degenerative disk disease, spondylolisthesis, ligamentous injury, and vertebral fractures. Additionally, the procedure has great utility in situations where emergent fixation is necessary as in trauma patients and in cases with contraindications to repositioning such as those with an exposed abdomen. By avoiding repositioning, operative time dropped significantly and intraoperative blood loss was comparable. Consequently, in select patients with adequate sized pedicles, performing simultaneous procedures offers an advantage over sequential surgery requiring repositioning. Implementing the operations together accomplished a three-column fusion with increased stability over each procedure performed alone. Patient outcomes were excellent and comparable to procedures done in series. We conclude that the lateral interbody fusion and percutaneous pedicle screw procedures are both readily accomplished in the lateral decubitus position and our preliminary data of this new method indicates that it offers less operative time and a promising potential reduction in morbidities.

With modern technological advances in surgical techniques, imaging modalities, bone graft alternatives, and attempts to decrease patient down time, spine surgery has seen a tremendous shift into the realm of minimally invasive surgery over the last 20 years [1–3, 9]. From the introduction of the operating microscope for discectomies to the more recent transformation of interbody fusions into the LIF, spine surgeons have adopted the notion of minimally invasive techniques. Attaining complete arthrodesis has been at the center of these recent advances in spine surgery, and introduction of bone morphogenic protein (BMP) and the interbody cage are just examples towards this aim [3]. In the realm of lumbar interbody fusions, the development of percutaneous pedicle and facet screw placement by Magerl would set the stage for Mayer, McAfee, and Pimenta for development of minimally invasive interbody fusion methods [2, 21, 36]. The novelties to this operation keep emerging with the most recent addition by Le et al. and Wang et al. to include a lateral plate [8]. Here, we introduce another innovative modification that would offer advantages to both the surgeon and the patient.

The technique described in this paper is to be considered for select patients undergoing a minimally invasive lateral transpsoas interbody fusion approach with a concomitant posterior percutaneous instrumentation. Our recommendation on the feasibility of this technique for a given patient is primarily determined by patient's bony anatomy, body habitus, and the number of levels fused. Pedicles for instrumentation should be large and clearly visualized on radiographs. Additionally, imaging of this anatomy may be affected abundance of soft tissue, a limitation in obese patients. Finally, the benefits of the procedure are not appreciated beyond two levels of instrumentation, and thus we do not recommend it.

A limitation of this study is that all pedicle screws that were placed in the lateral position were performed at a single center; thus the results need to be confirmed in a multicenter study. It is theoretical that the learning curve of this technique is commensurate with surgeon experience and that the combination of the senior surgeon's advanced experience and efficient workflow may explain the comparable excellent outcomes. In addition, there was no comparative radiographic review of lordosis between the repositioned and nonrepositioned patients. In that realm, a recent review by Yson et al. questioned whether prone-repositioning was necessary to gain the needed lordosis from posterior fixation following LIF [37]. In over fifty LIFs, they concluded that posterior fixation could be performed in a lateral position as there was no lordosis gained from repositioning.

Simultaneous lateral interbody fusion and percutaneous posterior pedicle screw fixation avoided repositioning, shortened operative time. Combining both procedures into a single lateral position also maintains surgical precision with comparable excellent outcomes. We are hopeful that this novel technique will contribute to the advancement of modern, minimally invasive spine surgery.

## 8. Conclusions

The lateral interbody fusion and percutaneous pedicle screw procedures can both be accomplished in the lateral decubitus position. In select patients with adequate size pedicles, performing simultaneous procedures offers an advantage over sequential surgery requiring repositioning. Performing the surgeries together accomplished a circumferential fusion with increased stability over each procedure performed alone. Patient outcomes were excellent and comparable to procedures done in series.

## Figures and Tables

**Table 1:** Characteristics of patients with repositioned versus nonrepositioned surgery.

Characteristic	Population	
	Repositioned	Nonrepositioned
Number of patients	10	10
Mean age (range)	57.8 (45–71)	54.5 (30–78)
Gender		
Male	4	7
Female	6	3
M/F ratio	1 : 1.5	2.3 : 1
BMI, mean	28.46	24.87
Weight (kg), mean	82.9	75.64
Pertinent history (%)		
Pars fractures	2 (20)	2 (20)
Spondylolisthesis	2 (20)	2 (20)
Severe degenerative disc disease	6 (60)	6 (60)
Operative time (minutes), mean	190.3	130.5^*∗*^
Estimated blood loss (mL), mean	93	108
Days hospitalized	4.1	3.8

^*∗*^ *p* < 0.05.

## References

[1] Oppenheimer J. H., DeCastro I., McDonnell D. E. (2009). Minimally invasive spine technology and minimally invasive spine surgery: a historical review. *Neurosurgical Focus*.

[2] Shen F. H., Samartzis D., Khanna A. J., Anderson D. G. (2007). Minimally Invasive Techniques for Lumbar Interbody Fusions. *Orthopedic Clinics of North America*.

[3] Mayer H. M. (1997). A new microsurgical technique for minimally invasive anterior lumbar interbody fusion. *Spine*.

[4] McAfee P. C., Regan J. J., Peter Geis W., Fedder I. L. (1998). Minimally invasive anterior retroperitoneal approach to the lumbar spine. Emphasis on the lateral BAK. *Spine*.

[5] Ozgur B. M., Aryan H. E., Pimenta L., Taylor W. R. (2006). Extreme Lateral Interbody Fusion (XLIF): a novel surgical technique for anterior lumbar interbody fusion. *Spine Journal*.

[6] Ahmadian A., Deukmedjian A. R., Abel N., Dakwar E., Uribe J. S. (2013). Analysis of lumbar plexopathies and nerve injury after lateral retroperitoneal transpsoas approach: diagnostic standardization. *Journal of Neurosurgery: Spine*.

[7] Le T. V., Smith D. A., Greenberg M. S., Dakwar E., Baaj A. A., Uribe J. S. (2012). Complications of lateral plating in the minimally invasive lateral transpsoas approach. *Journal of Neurosurgery: Spine*.

[8] Anand N., Baron E. M., Bridwell K. H., DeWald R. L. (2001). Direct lateral approach to the lumbar spine. *Textbook of spine Surgery*.

[9] Le T. V., Burkett C. J., Deukmedjian A. R., Uribe J. S. (2013). Postoperative lumbar plexus injury after lumbar retroperitoneal transpsoas minimally invasive lateral interbody fusion. *Spine*.

[10] Anand N., Hamilton J. F., Perri B., Miraliakbar H., Goldstein T. (2006). Cantilever TLIF with structural allograft and RhBMP2 for correction and maintenance of segmental sagittal lordosis: long-term clinical, radiographic, and functional outcome. *Spine*.

[11] Christensen F. B., Hansen E. S., Eiskjær S. P. (2002). Circumferential lumbar spinal fusion with brantigan cage versus posterolateral fusion with titanium cotrel-dubousset instrumentation: a prospective, randomized clinical study of 146 patients. *Spine*.

[12] DeBerard M. S., Colledge A. L., Masters K. S., Schleusener R. L., Schlegel J. D. (2002). Outcomes of posterolateral versus BAK titanium cage interbody lumbar fusion in injured workers: a retrospective cohort study. *Journal of the Southern Orthopaedic Association*.

[13] Yashiro K., Homma T., Hokari Y., Katsumi Y., Okumura H., Hirano A. (1991). The Steffee variable screw placement system using different methods of bone grafting. *Spine*.

[14] Burns B. H. (1933). An operation for spondylolisthesis. *The Lancet*.

[15] Enker P., Steffee A. D. (1994). Interbody fusion and instrumentation. *Clinical Orthopaedics and Related Research*.

[16] Karim A., Mukherjee D., Ankem M., Gonzalez-Cruz J., Smith D., Nanda A. (2006). Augmentation of anterior lumbar interbody fusion with anterior pedicle screw fixation: demonstration of novel constructs and evaluation of biomechanical stability in cadaveric specimens. *Neurosurgery*.

[17] Kozak J. A., Heilman A. E., O'Brien J. P. (1994). Anterior lumbar fusion options. Technique and graft materials. *Clinical Orthopaedics and Related Research*.

[18] Bohm H., Harms J., Donk R., Zielke K. (1990). Correction and stabilization of angular kyphosis. *Clinical Orthopaedics and Related Research*.

[19] Bradford D. S. (1988). Adult scoliosis. Current concepts of treatment. *Clinical Orthopaedics and Related Research*.

[20] Gertzbein S. D., Court-Brown C. M., Jacobs R. R. (1988). Decompression and circumferential stabilization of unstable spinal fractures. *Spine*.

[21] Drazin D., Liu J. C., Acosta F. L. (2013). CT navigated lateral interbody fusion. *Journal of Clinical Neuroscience*.

[22] Cloward R. B. (1953). The treatment of ruptured lumbar intervertebral discs by vertebral body fusion. I. Indications, operative technique, after care. *Journal of Neurosurgery*.

[23] Ntoukas V., Müller A. (2010). Minimally invasive approach versus traditional open approach for one level posterior lumbar interbody fusion. *Minimally Invasive Neurosurgery*.

[24] Faciszewski T., Winter R. B., Lonstein J. E., Denis F., Johnson L. (1995). The surgical and medical perioperative complications of anterior spinal fusion surgery in the thoracic and lumbar spine in adults. A review of 1223 procedures. *Spine*.

[25] Hee H. T., Castro F. P., Majd M. E., Holt R. T., Myers L. (2001). Anterior/posterior lumbar fusion versus transforaminal lumbar interbody fusion: analysis of complications and predictive factors. *Journal of Spinal Disorders*.

[26] Humphreys S. C., Hodges S. D., Patwardhan A. G., Eck J. C., Murphy R. B., Covington L. A. (2001). Comparison of posterior and transforaminal approaches to lumbar interbody fusion. *Spine*.

[27] Rosenberg W. S., Mummaneni P. V. (2001). Transforaminal lumbar interbody fusion: technique, complications, and early results. *Neurosurgery*.

[28] Foley K. T., Gupta S. K. (2002). Percutaneous pedicle screw fixation of the lumbar spine: preliminary clinical results. *Journal of Neurosurgery*.

[29] Holly L. T., Schwender J. D., Rouben D. P., Foley K. T. (2006). Minimally invasive transforaminal lumbar interbody fusion: indications, technique, and complications. *Neurosurgical Focus*.

[30] Isaacs R. E., Podichetty V. K., Santiago P. (2005). Minimally invasive microendoscopy-assisted transforaminal lumbar interbody fusion with instrumentation. *Journal of Neurosurgery: Spine*.

[31] Hackenberg L., Halm H., Bullmann V., Vieth V., Schneider M., Liljenqvist U. (2005). Transforaminal lumbar interbody fusion: a safe technique with satisfactory three to five year results. *European Spine Journal*.

[32] Hannon J. K., Faircloth W. B., Lane D. R. (2000). Comparison of insufflation vs retractional technique for laparoscopic- assisted intervertebral fusion of the lumbar spine. *Surgical Endoscopy*.

[33] McAfee P. C., Bohlman H. H., Yuan H. A. (1985). Anterior decompression of traumatic thoracolumbar fractures with incomplete neurological deficit using a retroperitoneal approach. *The Journal of Bone & Joint Surgery—American Volume*.

[34] Hood B., Vanni S., Chung K. J. (2012). Minimally invasive extreme lateral trans-psoas approach to the lumbar spine: applications and techniques. *Spine Surgery*.

[35] Knight R. Q., Schwaegler P., Hanscom D., Roh J. (2009). Direct lateral lumbar interbody fusion for degenerative conditions: early complication profile. *Journal of Spinal Disorders and Techniques*.

[36] Magerl F., Uhthoff H. K., Stahl E. (1982). External skeletal fixation of the lower thoracic and the lumbar spine. *Current Concepts of External Fixation of Fractures*.

[37] Yson S. C., Sembrano J. N., Santos E. R. (2012). Does prone repositioning before posterior fixation produce greater lordosis in lateral lumbar interbody fusion (LLIF)?. *Journal of Spinal Disorders & Techniques*.

